# Some Chemical Aspects of Neurosis

**Published:** 1901-01-15

**Authors:** J. S. Cassidy

**Affiliations:** Covington, Ky.


### SOME CHEMICAL ASPECTS OF NEUROSIS.

BY J. S. CASSIDY, M.D., D.D.S., COVINGTON, KY.

Read before the Ohio State Dental Society, December, 1900.

When chemistry, directed by human intelligence, lays its relentless hand on organized structure, for purposes of analysis, the identity of that structure is forever lost.

To prove the truth of this statement we need go no further than the vestibule of dental science, and select a tooth as a simple example for investigation. In building up this simple organ, vitality governs the selective affinities of the nutrient radicals, with as much care as it does in the formation of any other tissue; and when this organ parts from the source of life, it still retains the impress of the vital force; it is still organized. We can agree with Buzelius and others, that the constituent materials of this tooth as a whole consist of 28 percent of so-called organic substance, and 72 percent of calcium phosphate and other so-called inorganic salts. But in order to ascertain these results by analysis, the complete destruction of the tooth itself must precede the determination of the fact.

The chemist may tell in advance the effect, if any, on a given substance, by, for instance, prussic acid; no one, however, so far as I know, can detect the immediate chemical reactions that possibly occur when this acid is introduced into the animal body.

Realizing that, although the laws of chemistry are not violated by vitality, the vital force says to man, with his tests, tubes and reagents, "Hands off; thus far you may go, but no farther." Is it any wonder, therefore, that the present writer hesitated, with fear and trembling, the order to obey the command of your indefatigable executive chairman, to write a paper for this meeting on such a stupendous subject as the "Chemical Aspects of Neurosis"?

Taking it for granted that we understand, more or less, the general anatomy and physiology of the nervous system, we need not devote any time to their consideration; two or three explanations, however, will not be out of place.

A psychosis is a morbid mental state. This includes various conditions, such as insanity, idiocy, imbecility, morbid fears, mental irritability, depression, emotional excitement, volitional weakness, lack of self-control, weakness of memory and a tendency to hypnotic and somnambulistic states; also certain cerebral symptoms, as stupor, coma and some forms of headache.

Neurosis is a morbid, nervous state; not restricted, however, to morbid, nervous state of functional character. A combination of mental and nervous symptoms form psycho-neurosis, and all of these may be both, or either, subjective or objective.—*Dana.*

The chemistry of cell metabolism, constructive and destructive, is now, and, in the nature of the case, probably always will be, beyond the reach of satisfactory direct human examination; but animal and vegetable chemistry has not been restricted in the successful investigation of the nature of the secretory and excretory products, and of the proximate principles of the various tissues. Much has been accomplished in this direction that is suggestive of further interesting developments and a still deeper insight into the more complex molecules. The proteids, for example, which form the chief part of the solid portion of blood, muscles, nerves, glands and other organs, and which animals obtain direct from the vegetable kingdom, are composed approximately of $C_{54}$ $H_{7}$ $N_{17}$ $O_{23}$ $S_{2}$; some also contain phosphorus. Proteids, when heated to a temperature of 150° C. give off relative quantities of $NH_3$ and $CO_2$, corresponding exactly with those obtained by resolution of urea, $CO(NH_2)_2$, into ammonia and carbon dioxid.

This fact tends to show that albumen is a complex ureid containing one-fifth of its N in the form of urea, a substance metameric with ammonium cyanate ($NH_4CNO$). On the other hand, it is believed by some late authorities that proteids are built up by a series of cyanic alcohols, embodied on a benzine nucleus.—*Fowne.*

May it not be possible, that inasmuch as N, when present as a constituent of organic compounds, enters the combination with two units less than its normal valency, and therefore induces some influence on the retrograde metamorphosis of proteids; and the development of leucomains in health, and ptomains in disease, and also the rapid action of alkaloids in general when taken into the body?

By average results of various experiments, it has been found that the proportion of $CO_2$ exhaled to the O inhaled, is much greater in the day than in the night; with perfect rest day and night, twice as much, with active motion during the day, three times as much. The amount of O taken in during rest by day is only half as much as is taken in at night, and after active motion during the day the amount of O taken at night is still more.—*Kirke.*

In fevers the amount of $CO_2$ exhaled, proportional to the amount of O inhaled is much greater than in health; but in diabetis, the proportion of $CO_2$ exhaled by day to the O inhaled is less than in health, and at night the amount of O inhaled may be less than half the quantity required in a state of health. In leucomia, the proportion of $CO_2$ exhaled to O inhaled by day, was much less than in health, and the amount of O taken at night was even less than is taken in during the day.

These facts, obtained through governmental patronage, prove at least that O is stored up in the body during periods of rest, and that this O of reserve is frequently supplemented by the constitutional O of the tissues, and also that the relative amount of O inhaled plays a most important part in the rise and fall of animal vitality.

Nervous tissue are alkaline in reaction, but become acid by active work, the acidity is due to lactic acid ($C3H6O3$), and some uric acid ($C5H4N4O3$), greater in the gray matter than elsewhere.—*Dana.*

Of the inorganic constituents of the brain, aside from water, those of phosphorus are the most abundant, those of potassium next.—*Leoghegan.*

Proteids make up a little more than one-half the solids in gray matter, one-fourth those in white matter, and one-third those in nerves. The gray matter contains twice as much phosphorus as the white, while the white matter contains 52 percent of the monatomic alcohol, cholesterin and fat, and the gray matter only 18½ percent.

Cerebrin ($C17H33NO3$) is found largely in the brain (cerebrum), and licithin ($C42H84NPO9$), although occurring especially in the cranial nerves, is widely diffused in the animal organism. These two compounds form the so-called "protogon," which is the chief substance in the nerve centers and periphery.

Now, as neuroses are involved in pretty much all forms of disease, it is plainly impossible to take up more than a few salient points, here and there, such as might seem to agree with the subject imposed on your program.

Palpitation of the heart may be one of these, a symptom produced by various causes, reflex and direct. The heart itself is said to be a muscle, but with Whittaker we may ask, "What is muscular tissue anyhow but the terminal expansion of nerve fiber?"

One of the most frequent causes of palpitation of the heart are the chemical causes, at the head of which stands poisoning by nicotine. (Tea, coffee and alcohol are also in this chemical class). Tobacco smokers form a large contingent of cases of heart neurosis. What chemical action nicotine performs in the body is unknown, but it is known that when disturbance shall have once occurred as a result of the excessive use of tobacco, even a moderate use will suffice to keep it up.—*Whittaker.*

Although the baneful influence of nicotine is conceded as a prolific chemical cause of cardiac neurosis, the well marked injury to the whole nervous system observed in the chronic condition of persistent cigarette fiends must be attributed to other chemical reactions than are due to this alkaloid. The conclusions reached by Dudley after careful experiments in this direction, with lower animals, are that nicotine plays only a subordinate part in producing the evils concomitant with the habit. He says it is the absorption into the blood of CO which causes the greater injury. As is known $CO_2$ is one of the products of the combustion of tobacco. $CO_2$ formed at the burning end of the cigarette instead of escaping into the air, is drawn back into contact with the heated carbon, and there suffers reduction, according to the equation.

$$CO_2 + C = 2CO.$$

CO is a gas slightly lighter than air, colorless, tasteless and without odor. It is an unsatisfied dyad radical, anxious for more O, with which it takes up readily, when permitted. It is the insidious poison that escapes through the heated cast iron of our stoves and furnaces into the atmosphere of our rooms, and there, whether in palace or cabin, even in the relatively small quantity of one part to ten thousand of air, exerts its silent influence against the health of unsuspecting people, inducing serious neurosis, such as heart palpitation, headache, loss of appetite, anemia and systemic ennervation, particularly at the close of a long, cold winter.

When this poison is taken into the lungs by inhalation, in company with the smoke of the cigarette, it enters the circulation and attacks the red corpuscles, causing them to part with their hemoglobin, much like the physical effect on them produced by chloroform, but in this case appropriating the oxygen; and in so doing exerting a stimulating effect, followed by mental and physical depression; a true psycho-neurosis.

Some of the paper wrappers of cigarettes have been found to contain arsenic. This drug of itself might cause neurosis. While the immediate chemical action on tissue in rapid poisoning by arsenic is not fully determined, chronic poisoning by arsenic, however, shows a wholesale destruction of proteids and fatty degeneration; even if the arsenic took away only the sulphur of the proteids, the latter would, of course, be totally destroyed.

Alcohol, like CO, is a combustible, and being so, in the animal body as elsewhere, it seeks and finds oxygen. The O of reserve in the body is called upon, and willingly responds; if excess of alcohol is present the O of the proteides, rather than that of the ethereal salts, is also called upon for the alcoholic equivalent. Thus, both the principal and interest of the investment are used to support the chemical riots in the body of the victim, temperature, mental and physical power are diminished by the proteon waste, in undue proportion to the temporary exaltation of the early stages of indulgence. The various neuroses caused by alcoholism are too well known to justify mentioning here, but it is safe to say that if the habit be persisted in to the point of over-indulgence, bankruptcy of the whole nervous system is only a question of time.

A person in a state of health, free from bad habits and untoward circumstances, will furnish saliva usually alkaline. This alkalinity increases during and after eating, and decreases by experimental fasting; the writer thinks he has observed that fasting enforced by lack of appetite through excessive grief is accompanied by increase in the alkalinity of saliva, due, probably, to the extra development of ammonia from the proteids, and its combination with carbonic acid. Circumstances, however, may change this alkaline condition to a decided acid tendency, as is found with such diseases as catarrh of the intestinal tract, diabetis and acute rheumatism. Saliva may show a local acid reaction, caused by fermentation of food debris in the mouth, although at the same time it may be secreted alkaline. Whenever it comes acid from the ducts, we may presume on the existence of some neurosis, and in either case bacterial influence may be considered necessary. In the opinion of the writer, bacterial influence is not independent of that exerted by the vital force, and that chemical changes in the mouth, as elsewhere in the body, are undoubtedly governed by the rise and fall in the regulating power of the life principle; in other words, the presence of what is regarded as a purely local disease of whatever kind in the mouth, or elsewhere in the body, is owing to a combination of both local and systemic phenomena.

We have all seen, not infrequently, caries of the teeth, after progressing rapidly for a time, completely arrested, and immunity from further progress continued for many years, although at the same time the local environment may appear as in every way a most inviting trysting place for prolific and continuous development of bacterial ferments. On the other hand, we often observe that caries proceeds rapidly in otherwise healthy and cleanly mouths. While these reversed conditions are not by any means the rule, they occur among all classes of people of every age so frequently as to show to the minds of not a few that dental caries is a disease consequent on some neurosis that permits the local play of chemical affinities more or less destructive to the teeth at certain periods. Has nature so impressed itself upon these organs, in pairs, right and left, that simultaneous decay occurs on corresponding surfaces during the same space of time, leaving their immediate neighbors unaffected, although surrounded, apparently, by similar extraneous conditions? An answer affirmative to this question is not contrary to the facts obtained by clinical observation.

Of late years, leading writers in our profession assume the belief that structural perfection, or imperfection, density, hardness, or relative softness, in enamel or dentin, have little, perhaps nothing, to do in either resisting, or submitting to, the action of those local agencies to whose power the existence of dental disease is supposed to be attributed. Can it be that the influences for either good or evil, which induce these opposite phenomena, are the result of odic, nervous or electrolytic force, or whatever combination of forces it may be called, pertaining to and following the lines of some periodic rule involved in the local issue?

It is not sufficiently satisfying to be told what everybody knows, because told so often, that heredity, that vague, indefinite impression for the past, recent and remote is a recognized factor in influencing immunity from or encouraging susceptibility to certain diseases.

A state of health should not alone consist in the mere absence of disease, and as there are recognized odors peculiar to many diseases, so also there ought to be a positive, tangible evidence, belonging to and emenating from the healthy body, which our olfactory nerves are not as yet capable of detecting, but which, it seems, our dogs easily identify hours after our bodies have passed on the trail.

Will chemistry some day be able to isolate this mysterious material principle of health? "Quen Sabe."

With these thoughts in view, not as well expressed as they should have been, but nevertheless too real to be classed as mere theories, it is not perhaps presuming too much to say, that in connection with local cleanliness as an adjuvans, regular systemic treatment in each case will enter as the largest item in the future story of dental prophylactic therapeutics.

DISCUSSION OF DR. CASSIDY'S PAPER.

W. A. PRICE: The paper is too comprehensive for a proper discussion on such short notice; I have, therefore, no direct criticisms to make, but simply two points to make in brief.

The first is the great importance of appreciating the relation of the constitutional conditions to the changes which take place in the teeth. And if we are to understand and appreciate these, it follows that we must know the chemistry and physiological chemistry of all the organs of the body. The other point, the saliva, if acid as secreted by the duct, invariably indicates a neurosis. That is fairly well established. If we conclude that the patient is suffering from some form of neurosis, when such conditions exist, we have the key to the treatment, which to my mind, is just as important to the patient as the stopping of a cavity. The question then comes up, can neurosis be cured by constitutional treatment? Can the future dentist by the study of physiological chemistry, by laboratory experimentation, develop to such a point that he will have a course of treatment for such patients, so the neurosis can be treated properly, and thus re-establish the natural alkalinito of the mouth?

DR. H. T. SMITH: A development of the theories presented by Dr. Cassidy will certainly have a great effect upon the practice of dentistry and the treatment put into practice and recommended to patients.

The interest in physiological chemistry is of recent origin; as is noticed in the recent establishment of such laboratories at Harvard, and even in our own city for the purpose of experimental work. Dr. Cassidy's explanation of the immunity of certain teeth from caries, and occurrence and re-occurrence of caries in the mouth, is very nicely brought out, and I am very glad to have heard it.

The point to which Dr. Price has called attention, the re-establishing of the alkalinity of the saliva as a cure for caries of the teeth, is one which is still unsolved and under discussion. It would seem a very easy matter to prevent caries of the teeth by re-establishing the natural alkalinity of the saliva, by the use of various mouth washes, etc., but yet it is not the case; they do not do it. Re-establishment by systemic treatment, it seems to me, is a much likelier method of approaching to a cure of dental caries.

Dr. Fletcher: The statement with which the paper opens interested me very greatly. The chemistry of organized life is almost beyond our capacity for handling; it brings to our minds the experience of physicians in treating these diseases: it brings us to the matter of how emperical our knowledge and methods of treatment are.

As to the effects of systemic treatment upon the conditions of the mouth. I think this paper is of the greatest interest to every one of us. The idea of testing the saliva to diagnose systemic or pathological conditions is something which could be worked out to the very great advantage of our patients. We have in our hands as suggested by our essayist, theories to work upon, simple, plain directions as to how to benefit our patients. It goes without saying that the destruction of the lime salts of the teeth must be due to an acid. The suggestion made, that the saliva being acid when secreted is probably due to systemic conditions, or pathological nervous conditions, indicates the lines upon which we should investigate.

Dr. C. M. Wright: When Dr. Cassidy gave me the title of his paper, I wondered how my learned friend would treat this profound subject—in what manner he would view it. I can not quite appreciate his manner of handling it, I must say; but I am very much pleased with it; it is a monumental paper, worthy of this Society and of Dr. Cassidy.

I have long known that there is a chemical aspect of every disease, just as there are chemical aspects of every physiological phenomenon; there is a chemical aspect—there must be—in the metabolism in the blade of grass, just as there is a chemical aspect in the metabolism, anabolic and catabolic in the neuron cell.

It is interesting to note the advance made by neurologists within the last ten years in studying cellular biology, and the facts they have discovered. They view each nerve cell as a nerve organism, taking in food and giving off waste matter; it takes the food from the plasma of the blood. They have also described the changes which take place in the cell when the plasma of the blood is interfered with in any way. They study also the waste products of these nerve cells, and they have found that the nucleus escapes and is carried out. This causes a vitiated current of the blood, the blood being effected by the lymph, the latter in turn having taken up the waste products of the nerve cell, and thus reflexly causing neurosis.

I do not know whether this means a disease of the nerve cells, or whether it is a manifestation of interrupted or disordered nerve action; at any rate, I think we may consider when we have effects or manifestations of this kind that there has been some disturbance, either in nutrition or in the lack of energy.

I was very much interested in the conclusion of one of the theories of Dr. Cassidy's paper. In regarding dental caries as the result of neurosis, I think it would be well to announce, as adopted here in Columbus in 1900, that dental caries is a neurosis—that is, that the primary cause is neurosis, and that it is not due directly or immediately to bacteriological effects, and we will begin to-day to treat it from that standpoint.

Dr. Barnes: I do not rise to discuss this paper scientifically, but when I asked Dr. Cassidy to write upon this subject I had in mind the very conclusion which he has given us here to-day. I have often said that dentistry of the future would include the study of neurotic affections. We have more trouble from this cause than any other source. I could name case after case. We should be able to determine the rise and fall of the disease, or neurosis, by the decay of the teeth.

For example, take caries of one side of the mouth and total immunity on the other; you have no doubt sometimes wondered what caused it. If you will question your patients from this time forth you will find that in 99 percent of these cases they rest upon that side of the face at night, and chemical action is going on all that time.

Another thought occurs to me: when we become fatigued we rest ourselves, and thus tone our muscles by systemic treatment, as it were, not by medicine, not by local applications or anything of that kind; we simply rest our bodies, and as we do this our nerve tone is increased.

Dr. S. D. Ruggles: One subject mentioned by Drs. Price and Smith, that the alkalinity of the saliva has a marked effect on the decay of teeth. Why would not the acid of the saliva have the tendency to reduce decay instead of assisting? It is well known that a marked amount of acid in the culture will retard the action of bacteria and prevent further growth. Since this decay is due to action of bacteria, why would not the acid destroy the bacteria and thus prevent decay? I have always been under the impression that an alkaline media will give a better growth of bacteria.

Dr. D. W. Clancey: I do not rise to discuss the paper —I do not feel qualified—but to give expression to my full appreciation; and, as one of the reasons why I am glad I am here, and I shall take greater pleasure in reading it over more carefully. I congratulate the Society on having such a paper read before it.

Dr. Cassidy: I do not wish to occupy any time in closing this discussion. I feel very thankful for the bouquets thrown at me. I think the discussion has quite balanced the paper.

In answer to the question raised by Dr. Ruggles, all bacteria secrete an acid which is a poison to themselves, but not necessarily to all other bacteria. For example, lactic acid will destroy the lactic acid ferments or germs, if present in any quantity. But in a mouth where the acid attacks the teeth the acid is neutralized in its action on the teeth; then the germs can go on multiplying and producing decay.

The point intended to be made in the statement that when the saliva comes from the ducts in an acid condition we may presume that it is caused by neurosis, but this does not mean we should neutralize that condition with local mouth washes. With all due respect to Dr. Fletcher's powder, which he introduced last year, this applied locally will have no permanent effect; we must look for a specific neurosis, which must be overcome by systemic treatment. I thank you all very kindly.

Sensative Teeth and Dental Operations.—Advise your patients to avoid acids and to use an alkaline wash for a couple of weeks before and while undergoing dental operations, and they will suffer much less from sensative dentin.—*C. C. Harris, Dental Cosmos.*

